# How long should young infants less than two months of age with moderate-mortality-risk signs of possible serious bacterial infection be hospitalised for? Study protocol for a randomised controlled trial from low- and middle-income countries

**DOI:** 10.7189/jogh.13.04056

**Published:** 2023-07-14

**Authors:** PSBI Study Group, Abdullah H Baqui, Mohammod Shahidullah, Bangabandhu Sheikh Mujib, Salahuddin Ahmed, Arunangshu Dutta Roy, Rasheda Khanam, Iffat Ara Jaben, Muhammad Shariful Islam, Sabina Ashrafee Lipi, Md Jahurul Islam, Manajjir Ali, Amha Mekasha, Abiy Seifu, Lulu Muhe, Damen Hailemariam, Bogale Worku, Solome Jebessa, Temsunaro Rongsen-Chandola, Nidhi Goyal, Amit Kumar, Nita Bhandari, Shayam Kaushik, Surjeet Kumar, Amitabh Jain, Mangla Sood, Rakesh Sharma, Jagjit Singh Dalal, Kundan Mittal, GP Kaushal, Vineeta Wadhwa, Vishwajeet Kumar, Aarti Kumar, Rashmi Kumar, Yashwant Kumar Rao, Ved Prakash, Vinay Pratap Singh, Pramod Kumar Singh, Vivek Kumar Singh, Shiv Kumar, Robinson Daniel Wammanda, Laila Hassan, Saraja Ahmodu Opaluwa, Ishaku Hassan, Aminu Shadrach Adamu, Bawa Ega, Benazir Baloch, Imran Nisar, Fyezah Jehan, Karim Manji, Christopher Robert Sudfeld, Rodrick Kisenge, Nahya Salim, Sarah Somji, Mohamed Kheri Bakari, George Kibogoyo, Kristina Lugangira, Veneranda M Ndensangia, Christopher Paul Duggan, Sachiyo Yoshida, Karen Edmond, Shamim A Qazi, Yasir Bin Nisar

## Abstract

**Background:**

Hospitalisation and a seven-day injectable antibiotics course are recommended by the World Health Organization (WHO) to treat suspected clinical neonatal sepsis / possible serious bacterial infection (PSBI). Some infants presenting with PSBI signs associated with a moderate risk of mortality may only need a two-day hospitalisation followed by outpatient care treatment with oral antibiotics to complete seven days of antibiotics.

**Methods:**

A multi-centre, individually randomised, open-label trial will be conducted in seven sites in six countries: Bangladesh, Ethiopia, India (two sites), Nigeria, Pakistan and Tanzania. A common protocol will be used with the same study design, including the participants, intervention, comparison, outcomes, quality control, and analysis procedures. 0-59 days old infants presenting with moderate-mortality risk signs (low body temperature (<35.5°C), movement only when stimulated, stopped feeding well) or two or more signs of clinical severe infection (CSI) will be assessed and pre-enrolled. After 48 hours of hospital stay, clinically stable infants with a negative C-reactive protein test will be randomised either to hospital discharge on oral amoxicillin (intervention) or continued hospitalisation (control) arm. The intervention arm will receive oral amoxicillin for five days, whereas the control arm will receive injection gentamicin plus injection ampicillin for five more days plus supportive therapy if needed. We plan to enrol 5250 eligible young infants, 2625 infants in each of the two study arms. An experienced, well-trained independent outcome assessor will visit all enrolled cases on days 4, 8 and 15 after the initiation of treatment to assess the study outcomes in both intervention and control arms. The primary outcome of poor clinical outcome defined as death between randomisation and day 15 of initiation of treatment, deterioration during the 7-day treatment period, or persistence of the presenting sign of CSI at the end of the 7-day treatment period will be compared to assess if an early discharge and outpatient treatment leads to superior or at least non-inferior clinical outcome than continued inpatient treatment. The harmonisation of activities, including methods and processes, will be carried out diligently. Central training will be conducted by the WHO coordinating team, a central data coordination centre to collate all data, standardisation exercises for all clinical signs and internal and external monitoring. All the selected sites have extensive research experience. Through regular online and physical meetings, data-based monitoring, and physical site visits by WHO monitors, quality assurance and harmonisation will be ensured. This trial has been approved by the WHO and local site institutional ethics committees.

**Discussion:**

If the results show that young infants with moderate-mortality risk PSBI signs can be safely and effectively treated on an outpatient basis after a shorter hospital stay, it will reduce the burden on the hospitals, potentially reduce nosocomial hospital infections and increase access to treatment for families with poor access to health facilities. It may also reduce the health system costs (human and materials) and allow the overburdened hospitals to pay more attention to critically ill young infants. In addition, this evidence will contribute to making a case for reviewing the WHO PSBI guideline.

**Registration:**

International Standard Randomised Controlled Trial Number, ISRCTN16872570.

Around 2.3 million neonatal deaths occurred in 2021 worldwide, accounting for 47% of under-five deaths [1], even though this burden has decreased recently. In South Asia and sub-Saharan Africa, neonatal infections account for over 35% of all neonatal deaths [2]. The World Health Organization (WHO) integrated management of childhood illness (IMCI) algorithm classifies neonates and young infants with clinically suspected sepsis as “Possible Serious Bacterial Infection (PSBI)” based on the recognition of seven clinical signs – fast breathing (60 breaths or more / minute) in 0-6 days old babies, severe chest indrawing, high body temperature (≥38^°^C), low body temperature (<35.5^°^C), not able to feed at all or stopped feeding well, convulsions, and movement only when stimulated or no movement at all among infants 0-59 days old [3]. WHO recommends that young infants with PSBI should be hospitalised and treated with injectable antibiotics and supportive care [4]. However, when referral to a hospital is not feasible, the WHO guideline recommends the further classification of these infants into those who are critically ill and those who have a clinical severe infection (CSI) (**Box 1**) [5]. Infants with CSI can be managed on an outpatient basis with a simplified regimen of injectable gentamicin for two or seven days and oral amoxicillin for seven days [5] based on clinical trials from Africa [6,7] and Asia [8,9].

Box 1 **Sub-classification of children with signs of possible serious bacterial infection when a referral is not feasible [5].**

Critical illness

• convulsions

• not able to feed at all

• no movement at all

Clinical Severe Infection

*Low-mortality risk signs*

• high body temperature (≥38°C*)

• severe chest indrawing

• fast breathing of ≥60 breaths per minute in <7 days old infants

*Moderate-mortality risk signs*

• low body temperature (<35.5°C*)

• movement only when stimulated

• not feeding well / stopped feeding well

*Thresholds based on axillary temperature.

Several countries have adopted the WHO PSBI guideline [5]. The safety and effectiveness of simplified outpatient therapy for PSBI have been shown through implementation research in several countries in Africa and Asia when hospitalisation is not feasible [10-21]. In the first two months of life, 8-10% of infants can have an episode of PSBI, whereas only 25-50% of them may reach a hospital for treatment in low and middle-income countries (LMICs). However, hospitalisation has innate risks, e.g. multi-drug resistant nosocomial infection [22-25], so only those sick young infants with PSBI signs should be hospitalised who have a favourable benefit-risk ratio. Secondary analysis of African Neonatal Sepsis Trial (AFRINEST) data [6,7] showed that those young infants with movement only on stimulation, not feeding well or low body temperature (<35.5^°^C) have a much higher case fatality risk (CFR) of 3.2%, 4.0% and 11.0%, respectively than those with high body temperature (>38^°^C), severe chest indrawing, or fast breathing (>60 breaths per minute in 0-6 days old infants), who had a CFR of 0.8%, 0.9% and 2.0%, respectively [26]. Infants presenting with two or more signs of CSI also had a relatively moderate risk of mortality (CFR = 5.7%). As expected, signs of critical illness were associated with a very high risk of death (convulsions (11.3%), unable to feed at all (22.9%) and no movements at all (25.0%)) [26].

An important inference from these findings is that infants with moderate mortality risk signs of CSI (stop feeding well, movements only on stimulation, low body temperature), multiple signs of CSI, as well as those with a critical illness [26] are likely to benefit from hospitalisation. In contrast, young infants with low mortality risk signs of CSI (severe chest indrawing, temperature >38^°^C or fast breathing with >60 breaths per minute in 0-6 days old) may not need inpatient treatment in a hospital. Additionally, analysis of AFRINEST data by place of treatment showed that CFR was three times higher (6.5%) in hospitalised young infants compared to those treated on an outpatient basis (1.9%) when they refused referral for the same signs of CSI [26]. This could be because they were either sicker, received sub-standard care at the hospital, or developed a nosocomial infection.

Of all young infants with any sign of CSI who received outpatient treatment in the AFRINEST study, 1.3% died up to 15 days after the initiation of treatment. Of those who died, 39.7% died within the first 48 hours of treatment. Among those who survived and were assessed at 48 hours of treatment, three-quarters of infants (73.9%) had no signs of illness. Of these infants who recovered by 48 hours, 0.4% died between day three and day 15. In contrast, mortality between day three and day 15 was higher among infants who still had the presenting sign at 48 hours (0.7%) or those who deteriorated (presence of new CSI sign / critical illness sign) at 48 hours (8.0%) (secondary analysis, unpublished data).

In high-income settings, infants with clinical suspicion of sepsis are hospitalised, and parenteral antibiotics are initiated after taking samples for sepsis screening and blood culture. After 48 hours, if the laboratory tests are negative and the infant is clinically well, antibiotics are stopped, and the infants are discharged [27-29]. However, this is hardly ever done in the LMICs, where once started, the parenteral antibiotics are continued for 7-10 days. We need evidence of whether sick young infants with moderate-mortality risk can be discharged early from the hospital.

The primary objective of this trial is to measure the effect of early discharge on an oral amoxicillin treatment on poor clinical outcomes compared with inpatient treatment in young infants 0-59 days old with moderate-mortality risk or two or more signs of CSI. We hypothesise that the majority of infants who improve by 48 hours will need shorter hospitalisation.

## METHODS

### Research question

Among hospital-admitted young infants 0-59 days old with any moderate-mortality risk sign or two or more low-mortality risk signs who clinically improve 48 hours after initiation of treatment and have a negative C-reactive protein (CRP) (population), are discharged from the hospital on oral amoxicillin at home for the next five days (intervention), compared with continued hospital management for next five days (control), non-inferior in terms of poor clinical outcome (outcome)?

### Study design, setting and sites

This will be an individually randomised, two-arm, open-label trial. The study will be conducted in seven sites in six countries Bangladesh, Ethiopia, India (two sites), Nigeria, Pakistan and Tanzania (**Table 1**).

**Table 1:** Details of the study sites*

Study site	Description
Bangladesh	The study will be conducted in four hospitals. Zakaiganj Upazila Health Complex (ZUHC), Zakiganj (primary / public), Sunamganj District Hospital (SDH), Sunamganj (secondary / public), Moulvibazar District Hospital (MDH), Moulovibazar (secondary / public), and Habiganj District Hospital (HDH), Habiganj (secondary / public) in Sylhet. ZUHC has six paediatric beds and one radiant warmer, SDH has 14 beds, one incubator and three warmers, MDH has 17 beds and 17 warmers, HDH has 11 beds and 11 warmers in their Neonatal Intensive Care Units (NICU) / Special Neonatal Care Units (SNCU) respectively. All are functional 24 hours a day. Except for ZUHC, all hospitals have trained paediatricians, but none have a neonatologist. Oxygen is available 24 hours a day through cylinders.
Ethiopia	The study will be conducted in five hospitals. One named Tirunesh Beijing Hospital (secondary / public) in Addis Ababa, and four in the Oromia region named Bishoftu Hospital (secondary / public), Adama Hospital (secondary / public), Batu Hospital (General Hospital), Asella Hospital, Asella (Referral Hospital). Tirunesh Beijing Hospital has 22 beds and 17 incubators / warmers, Bishoftu Hospital has 15 beds and 17 incubators / warmers, Adama Hospital has 53 beds and 11 incubators / warmers, Batu Hospital has 18 beds and two incubators / warmers and Asella Hospital 40 beds and eight incubators / warmers in their NICU/SNCU respectively. All are functional 24 hours a day. All hospitals have trained paediatricians, but none have a neonatologist. Oxygen is available 24 hours a day through cylinders.
Himachal Pradesh-NCR, India	The study will be conducted in five hospitals i.e. Dr YS Parmar Government Medical College Hospital (YSPGMC), Nahan (tertiary / public), Civil Hospital (CH), Poanta (secondary / public), Indira Gandhi Medical College Hospital (IGMC), Shimla (tertiary / public), Pt BD Sharma Post Graduate Institute of Medical Sciences (PGIMS), Rohtak (tertiary / public), Dr Baba Saheb Ambedkar Hospital (BSAH), Delhi (tertiary / public). YSPGMC has 25 beds and six incubators / warmers, CH has eight beds and eight warmers, IGMC has 24 beds and 10 incubators/warmers, PGIMS has 65 beds, and 38 incubators / warmers and BSAH has 42 beds and 34 incubators / warmers in their NICU / SNCUs respectively, and all are functional 24 hours a day. All hospitals have trained paediatricians, and only YSPGMC Nahan and PGIMS Rohtak hospitals have a neonatologist. Oxygen is available 24 hours a day through central supply.
Uttar Pradesh, India	The study will be conducted in three hospitals i.e. Hallet Hospital Kanpur (tertiary / public), Dufferin Hospital, Kanpur (secondary / public) and Shyam Children’s Charitable Hospital (SCCH), Kanpur (secondary / private). Hallet Hospital has 38 SNCU beds equipped with radiant warmers and 80 paediatric beds, Dufferin Hospital has 12 SNCU beds equipped with radiant warmers, and SCCH has 9 SNCU beds equipped with radiant warmers and 25 paediatric beds, and all are functional 24 hours a day. All hospitals have trained paediatricians, and none has a neonatologist. Oxygen is available 24 hours a day through central supply in Hallet Hospital and SCCH and cylinders in Dufferin Hospital.
Nigeria	The study will be carried out in three secondary / public hospitals i.e. Hajiya Gambo Sawaba General Hospital (HSGH), Zaria, Giwa General Hospital (GGH), Giwa and Yusuf Dantsoho Memorial Hospital (YDMH), Tudun Wada, Kaduna. HSGH has 14 beds and one warmer (one incubator is on order), GGH has 11 beds and one warmer whereas YDMH has 15 incubators, 11 beds and 14 warmers in their NICUs / SNCUs respectively. All are functional 24 hours a day. All hospitals are staffed by general-duty physicians. In addition, YDMH has a trained paediatrician / neonatologist. Oxygen is available 24 hours a day either through central supply, oxygen concentrators and cylinders at the Hajiya Gambo Sawaba General Hospital and Yusuf Dantsoho Memorial Hospital while only through cylinders at the Giwa General Hospital.
Pakistan	The study will be done in two secondary-level hospitals, i.e. The Aga Khan Hospital for Women and Children Hospital, Kharadar (AKU-KH), Karachi and Sindh Government Children Hospital (SGCH), and two tertiary care hospitals, i.e. National Institute of Child Health (NICH), and Sind Institute of Child Health (SICH), Karachi. AKU-KH has five cots, four incubators, and three radiant warmers. SGCH has 16 incubators, 8 cots, 8 beds in Paediatric ICU, and 110 beds in the paediatric ward. NICH has 67 incubators, 27 cots, 330 beds in the Paediatric ward, 55 beds in the emergency and 24 functional ventilators. SICH has 65 incubators, 20 ward beds and 14 ICUs with 21 ventilators. Central oxygen 24 hours a day and a radiant warmer are available. All four hospitals are functional with a facility of 24-hour emergency service. Qualified paediatricians and one neonatologist each staff all hospitals.
Tanzania	The study will be carried out in two public / secondary/regional referral hospitals i.e. Amana Hospital and Temeke Hospital. Amana Hospital has 59 cots and nine radiant warmers, whereas Temeke Hospital has 34 cots and seven radiant warmers in their NICUs / SNCUs, respectively. Both are functional 24 hours a day. Both hospitals are staffed by qualified paediatricians, and none has a neonatologist. Oxygen is available 24 hours a day through cylinders and oxygen concentrators.

*Additional hospitals may be included to increase enrolment depending upon the site-specific recruitment.

### Participants

All patients (<2 months old at presentation) admitted to the study hospitals with any moderate-mortality risk signs or two or more signs of CSI will be assessed for eligibility for this study 48 hours after initiation of treatment and considered for inclusion in the study if: 1) clinically well on day 3 (defined as the absence of all signs of critical illness or CSI (**Box 1**)) and 2) negative C-reactive protein and 3) the family lives within a catchment area where a follow-up of up to day 15 can be accomplished. Infants will be excluded from the study if they have any one of the following: 1) weight <2 kg at the time of presentation (if age at screening is less than 10 days) or weight-for-age <-3z (if age at screening is ≥10 days), or 2) signs of critical illness on admission (no movement at all, unable to feed at all, or convulsions), or 3) the appearance of any moderate-mortality risk sign or multiple low-mortality risk signs in the first 24 hours of life, or 4) hospitalised for any illness in the previous two weeks, or 5) prior use of injectable antibiotics in the last two days for the same illness (except the dose given for pre-referral administration), or 6) previously included in this study or currently included in any other study, or 7) any other reason to stay in the hospital, as decided by the treating physician.

Enrolled young infants who develop or are diagnosed with any new non-infectious problems after initiating antibiotics, such as jaundice, cardiac problems, etc. will be managed according to the hospital guidelines. They will not be considered to have poor outcomes.

Since some signs of PSBI mimic other conditions such as perinatal asphyxia, transient tachypnoea etc. it was decided during the investigators and Technical Advisory Group (TAG) meeting in March 2020 that newborns presenting with any sign of CSI within 24 hours of birth will not be eligible.

### C-reactive protein test

We will use a bedside, lateral flow chromatic immunoassay-based CRP test kit, Actim^©^ CRP test kit (Noljakante 13, F1-80130 Joensuu, Finland), that provides a semi-quantitative assessment of CRP. These will have been validated across all sites. A paediatric nurse will use a lancet to obtain a drop of blood from the fingertip or sole of the young infant with no signs of CSI or critical illness after 48 hours of hospitalisation using the WHO guidelines on drawing blood [30]. Consent for this sample will be taken at the time of screening. The sample will be processed as per kit instructions. A positive result can be read as soon as it becomes visible. One, two, and three blue lines correspond to a CRP of 10-40 milligrammes (mg) / litre (l), 40-80 mg / l, and >80 mg / l, respectively. A negative result, which corresponds to less than 10 mg / l of CRP, is indicated by the presence of only a red control line and should be confirmed at five minutes. Based on the results of the test, the treating physician will decide whether the young infant is eligible to be enrolled. There is no major risk associated with the test. However, bruise or mild soreness around the blood test site is common and can last for a few days.

Eligible infants will be enrolled if their parents provide informed written consent to participate in the study. They will be randomised to either continued hospitalisation for up to a total of seven days or discharged from the hospital on home treatment with an oral antibiotic for the next five days. Based on the findings of the systematic review (unpublished data), it was decided that a single, semi-quantitative (threshold level 10 mg / l) CRP will be performed 48 hours after admission for enrolment.

### Intervention

Discharge from the hospital and home treatment with oral amoxicillin for five days.

### Control

Continued injectable antibiotic treatment and supportive therapy in the hospital for seven days.

One of the possible reasons that outpatient care was observed to be better than hospital care in previous studies might be the poor quality of care in hospitals. In this study, we would like to reduce this factor as far as practically feasible. This means that the quality of care at the study hospitals will be reviewed against the WHO pocketbook for hospital care for children [4] and will be improved using quality improvement approaches to ensure a “minimum” quality of hospital care. Efforts to improve the quality of care will also be made at outpatient facilities.

### Outcomes

The primary outcome is “poor clinical outcome” defined as 1) death between randomisation (day three of initiation of therapy) and day 15 of initiation of therapy, or 2) presence of any sign of critical illness (no movement at all, unable to feed at all, or convulsions) or any sign suggestive of another serious infection, e.g. meningitis, bone or joint infection, on day four and eight of initiation of therapy, or 3) presence of any sign of CSI on day eight of initiation of therapy.

### Screening and enrolment

Young infants up to two months of age at the outpatient or emergency department will be examined, triaged, and stabilised by the consulting paediatrician or physician of the participating hospital. This process followed as part of regular facility procedures is referred to as “pre-screening”. If the paediatrician / physician observes signs of CSI during their initial assessment and the clinical condition of the infant is stable, the infant will be assessed by the study screening, enrolment and randomisation team. Screening of sick young infants will be performed by a study nurse / physician in the outpatient or emergency department of the participating hospital, and information will be captured on the case report form (screening and pre-enrolment form). Those who fulfil the above-mentioned inclusion criteria and do not have any exclusion criteria will be pre-enrolled after confirmation of clinical signs by the treating physician. The study nurse / physician will fill out the appropriate case report form at this stage. Infants who are not eligible for enrolment will be managed as per the treatment protocols of the hospital. All young infants with any moderate-mortality-risk sign or two or more signs of CSI (**Box 1**) will be admitted for injectable antibiotics by the treating physician. The CRP blood test will be conducted 48 hours post-admission. The study screening, enrolment and randomization team will re-screen the admitted infant using an appropriate case report form to assess the infant’s status and eligibility for the trial. Infants will be enrolled in the trial if they are clinically well at the time of the visit (none of the PSBI signs) and have a CRP <10 mg / l. However, if the treating physician has any concerns or reasons for wanting the child to remain in the hospital, then the child will not be considered for randomisation and will be excluded from the study.

**Figure 1:**
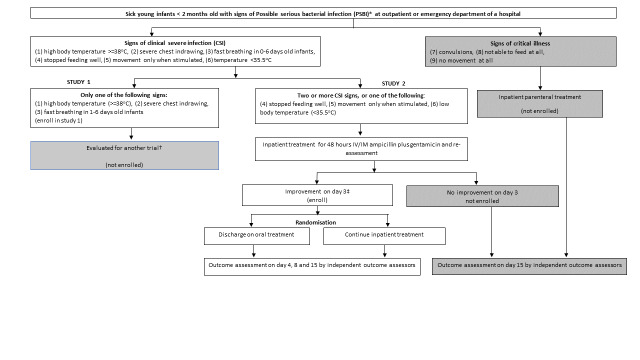
Study approach for screening, enrolment and management of sick infants. *Signs of possible serious bacterial infection (PSBI): convulsions, not able to feed at all, stopped feeding well, severe chest indrawing, body temperature ≥38°C, body temperature <35.5°C, movement only when stimulated, no movement at all, fast breathing in <7 days. †[31]. ‡Clinically well on day 3 defined as absence of all signs of critical illness or CSI, laboratory test negative, and family lives in a catchment area where a follow-up can be accomplished. CSI – clinical severe infection, IV – intravenous, IM – intramuscular, PSBI – possible serious bacterial infection

See the study approach for screening, enrolment and randomisation in **Figure 1** and the implementation strategy in **Table 2**. In addition, study information regarding objectives and procedures will be posted in the labour and delivery wards, antenatal care clinics and postnatal clinics in the participating hospitals.

**Table 2:** Implementation Strategy for Intervention and Control Arms

Activity	What	Who	When	Where	How
Case identification	Detection of cases	Screening, enrolment and randomisation team with hospital staff	The sick young infant is brought by the family for consultation	OPD or general or paediatric emergency department of the hospital	Clinical assessment
Screening, randomisation and enrolment	Detection of eligible cases	Screening, enrolment and randomisation team	The young infant is seen by the hospital staff and the study staff	OPD or general or paediatric emergency department of the hospital	Clinical assessment, administering informed consent, randomisation and enrolment
Hospitalisation	Admission and treatment with recommended WHO injectable antibiotics	Hospital staff	After pre-enrolment	Inpatient department	After clinical assessment and pre-enrolment
Laboratory test	C-Reactive Protein (CRP) testing	Hospital staff	48-72 hours after admission	Inpatient department	Bedside test using WHO provided kits
Eligibility for the trial	Assess infants’ status using an appropriate case report form	Screening, enrolment and randomisation team	48-72 hours after admission	Inpatient department	Checking the presence of clinical signs and CRP test results and obtaining informed consent before enrolment
Enrolment and randomisation	Enroll and randomise to the treatment arm	Screening, enrolment and randomisation team	48-72 hours after admission	Inpatient department	Using QR codes
Treatment provision (intervention arm)	Discharge from the hospital on oral amoxicillin	Mothers / caretakers will give oral antibiotic	After discharge from the hospital	At home on an outpatient basis	Practical demonstration of giving the first dose in front of the mother / caregiver
Treatment provision (control arm)	Continue hospitalisation for injectable antibiotics and supportive care	Hospital / study staff	After enrolment, consent and randomisation	Hospital	Giving injectable therapy
Treatment documentation	Follow-up for treatment documentation	Treatment documentation team	Days 4, 8 of initial admission	Intervention arm: patients' homes or OPD	Intervention arm: asking mothers and checking medicine bottles
				Control arm: hospital where the patient is admitted	Control arm: hospital inpatient treatment records
Supervision	Sub-sample on follow-up days for quality assurance	Study supervisor	Days 4, 8 and 15 of initial admission	Intervention arm: patients’ homes and OPD	Verification and substantiating assessment
				Control arm: hospital where the patient is admitted and patients’ homes	
Outcome assessment	Outcome assessment	Independent Outcome Assessment Team	Day 2, 4, 8, 15 post-randomisation and if the patient deteriorated in between	Intervention arm: patients’ homes or a hospital if the patient was admitted	Clinical assessment and filling outcome assessment form
				Control arm: hospital where the patient is admitted and at home after discharge	

OPD – outpatient department, CRP – C-reactive protein, CRF – case report form, WHO – World Health Organization

### Randomisation to treatment arms

WHO staff in Geneva not associated with site work prepared the randomisation list in blocks of four, six and eight and shared it with the Regional Triangle Institute (RTI), the study Data Coordination Centre (DCC). Allocation concealment was ensured using QR randomisation codes. Randomization was in a 1:1 ratio. RTI produced a list of encrypted QR codes and has these printed on labels based on the randomisation scheme for each study site. RTI transmitted the printed QR code blue colour labels to the sites.

Each hospital within a study site will have a single set of QR codes that all screening and enrolment team members must use, even if the screening and enrolment are taking place in different locations within the hospital. After determining eligibility, the data collector will take the QR code label next in the sequence from the list at their facility and scan this to obtain the randomisation number.

### Sample size

We will enrol a total of 5250 participants in the trial across study sites. Power calculations assumed 1:1 randomised, a one-sided type I error rate α of 0.025 (equivalent to a two-sided 95% confidence interval), and a loss to follow-up or withdrawal rate of 10%. The non-inferiority margin for the poor clinical outcome was selected as a relative risk of 1.40 for the outpatient group as compared to the standard inpatient group. This margin was selected based on the previous trials [6,7]. **Table 3** presents statistical power for trial outcomes varying the potential cumulative incidence of poor clinical outcomes in the standard inpatient group. We will have >88% power to test for non-inferiority if the cumulative incidence of poor clinical outcomes is 5-10% in the inpatient group.

**Table 3:** Statistical power calculations for incidence of poor clinical outcome

		Cumulative incidence of poor clinical outcome in standard inpatient arm		
		**10.0%**	**7.5%**	**5.0%**
Statistical power for poor clinical outcome	Noninferiority margin: Relative risk of 1.40	96.6%	97.5%	88.4%

### Dosage of medicines to be used

Infants randomised to the intervention arm will receive the first dose of oral amoxicillin in the hospital at discharge. Those randomised to the control arm will be in the hospital and receive injectable antibiotics (**Table 4**).

**Table 4:** Treatment arms

	Continuation of hospital treatment arm		Home arm
	Injection gentamicin	Injection ampicillin	Oral amoxicillin
Route	IM / IV	IM / IV	Oral
Frequency (per day)	Once	Two-four*	Twice
Dose by weight	Strength, 40 mg / ml	Strength 250 mg / 1.5 ml	250 mg dispersible tablet
1.5-2.4	0.2	0.8	1 / 2 tablet
2.5-3.9	0.4	1.2	1 / 2 tablet
4.0-5.9	0.6	1.5	1 tablet
Duration	Continue for a further 5 days (total 7 days)	Continue for a further 5 days (total 7 days)	Stop injections and start oral amoxicillin for 5 days

IM – intramuscular, IV – intravenous, mg – milligramme, ml – millilitre

*Depending on the age of the child, see [4], for the hospitalised child, twice daily in the first week of life, thrice daily in 2-4 weeks of life, four times daily in 4+ weeks of life.

### Treatment documentation

The study treatment documentation and compliance team will document the treatment received in the hospital (control) and home (intervention) from days one to seven after enrolment. For infants in the control arm, the documentation of treatment received will be captured from the hospital inpatient treatment charts. For infants in the intervention arm, the documentation of treatment received will be captured through mother reports or physicians' outpatient department (OPD) records.

### Outcome assessment

Due to the nature of the treatment, the trial cannot be blinded. To reduce potential measurement bias, we will use Independent Outcome Assessors (IOA) who will not be associated with the treatment of the infant. The IOAs who will be the Outcome Assessment Team members will conduct outcome assessment visits using the designated days four, eight and 15 post-admission. In the hospital arm, the outcome visits on day four will be done in the hospital, while on days eight and 15, visits will be done at home (or in the hospital or where ever the infant may have moved to). In the outpatient arm, the outcome visits on days four, eight, and 15 will be done at home or where ever the infant may have moved to. If the infant is not able to be seen on the specified day, then a window of additional time will be allowed for outcome follow up, i.e. for day four visit, an additional 24 hours; for day eight and day 15 visits, an additional 48 hours. The outcome team will continue to visit the family during this time window to complete these visits.

Outcomes will only be ascertained by the IOAs per the criteria mentioned above under outcomes. A case will be considered to have a poor clinical outcome if it is confirmed by the IOA.

### Follow-up

All infants will be followed up till day 15 after the initial admission. Infants in the intervention arm will be asked to return to the hospital for treatment and assessment by the hospital physician / nurse on day five of treatment initiation. Infants will be assessed for the presence of any adverse event during scheduled follow-up visits to the hospital OPD, hospital visits by the family for care-seeking at any time during the follow-up period, or reported to the IOA team during a home visit and referred for care. These will be managed by the treating physician as per routine practice and followed up till resolution.

Infants in the control arm will be followed up and managed as per routine hospital procedures.

All efforts will be made to follow all the enrolled young infants in both studies. If a young infant does not come to the hospital / health facility on the follow-up day, the study team will contact them by phone and counsel parents / caregivers to bring the infant to the hospital / health facility. Transportation costs will be provided to families of all patients if needed.

### Data handling and confidentiality

Data will be collected electronically using tablets. The safety and confidentiality of the data will be ensured. Data will be backed up regularly. Names or identities of participants will be unidentifiable and will not be used in any publication. Only the “research teams”, DCC and WHO will have access to data. The paper-based log books / registers will be retained for 10 years and then destroyed.

### Data management

RTI has developed the PSBI data management system using the Tangerine software platform (see http://www.tangerinecentral.org/ and https://docs.tangerinecentral.org/). RTI will run quality checks to identify missing data and will harmonise the data across sites, within sites and with WHO. It will provide monthly reports and data to WHO for administrative and supportive purposes. They will also provide data and frequency tables and trends to Data Safety Monitoring Board (DSMB) or the Technical Advisory Group (TAG) for overall monitoring purposes as required.

### Data analysis

Simple comparisons of means and proportions by intervention and control treatment arms will be carried out to evaluate the baseline comparability of the two groups. The Kolmogorov-Smirnov test of normality will be used to test the normality of the data. The primary analyses will be per-protocol to compare treatment outcomes by comparing proportions of poor clinical outcomes (including deaths and deterioration) between intervention and control arms using the Chi-square test, setting statistically significant as *P* <0.05. An intention-to-treat analysis will also be conducted. The clinical outcomes between control and intervention treatment arms will be compared, and the difference in the risk of poor clinical outcomes together with 95% confidence intervals, will be calculated. Secondary analyses will be performed to identify predictors of poor clinical outcomes.

### Quality control and assurance

All study teams (screening, enrolment, randomisation and outcome assessment teams) will have study supervisors who will support adherence to the manual of operations. The quality assurance team will conduct regular standardisation exercises, oversight and monitoring of all study activities through regular and random visits and checks of the proportion (10%) of all completed study forms. A site preparation review will be conducted before the initiation of the study. This will include the standardisation of practices and measurements. To ensure the quality of study implementation, external oversight and support will be provided by WHO staff and study consultants.

### Quality of care in hospitals

The hospital infant care services will be strengthened regarding human resources and capacity building through training, processes and standard operating procedures (SOPs), ensuring continuous availability of standard quality antibiotics and basic support for routine care. The “minimum” quality of care will be according to the WHO pocketbook for hospital care for children [4], which includes keeping the baby warm and providing kangaroo mother care to prevent hypothermia, encouraging a mother to breastfeed frequently to prevent hypoglycaemia, fluid management when required, basic laboratory support, and oxygen therapy when needed [4]. The hospital team will be oriented on the study protocol and recommended treatment protocols, including information on indications for changing the treatment regime. Regular visits to assess the quality of care at the hospitals will be made by an experienced paediatrician / neonatologist. Medical equipment provided to the participating hospitals for the study will remain there after the completion of the study.

### Training of health workers and study staff

The study staff who will perform screening, enrolment, and outcome measurement will be trained using a standardised study Manual of Operations. Data will be collected by research staff on standardised data collection forms. During training, emphasis will be placed on maintaining Good Clinical Practice (GCP) standards. All research staff will be trained in rapport-building and communication with mothers / caregivers. The screening and enrolment study staff will be trained to introduce the study to potential participants, administer the consent form, assess for clinical signs and eligibility, and correctly complete all relevant study forms. The outcome measurement staff will be trained in the definitions of outcomes and on their standardised assessment, as well as completion of the outcome assessment forms.

### Standardisation and refresher training

Periodic standardisation exercises every quarter will be carried out for study staff, supervisors and IOAs to the identification of clinical signs to maintain their clinical skills. The study sites will also develop a system of periodic refresher training for health workers, supervisors and IOAs through clinical practice and / or video demonstrations. In addition, periodic centralised standardisation exercises and refresher training will also be conducted by WHO for the study staff.

### Logistics and commodities

All sites will be equipped with necessary medicines, equipment and logistics for pneumonia case management, such as oral amoxicillin 250 mg (or 125 mg) dispersible tablets, pulse oximeter with accessories, digital and mercury thermometers, digital weighing scales, respiratory rate counting timers / digital timers and other necessary materials centrally through the WHO supply division to maintain standardisation. The study teams will monitor the equipment for periodic standardisation and replacement of faulty devices, if any. The consumables will be replenished as per need.

### Supervision

A study supervisor will supervise all study staff. A supervisor will periodically validate a proportion of young infants who are assessed as potential enrolment to ensure quality. Supervised accompanied visits and independent unaccompanied visits will be carried out regularly by the supervisors. Video calls or video clips of signs, e.g. severe chest indrawing, will also be used in real-time to allow supervisors to monitor the correct identification of signs from time to time. Principal investigators and co-investigators will also make random visits to check the performance and quality of the trial.

Periodic meetings of the research implementation teams will be held to review enrolment, performance, observations and follow-up problems and identify solutions / required actions to overcome those. Action points will be followed up on in the next meeting.

### Monitoring

All the sites will submit monthly progress reports to the coordinating site at the WHO, which will be reviewed, and feedback will be provided. Regular conference calls will be held with all sites to discuss the progress and critical issues of the study. Data-based monitoring, as well as verification of serious adverse event (SAE) forms, will be carried out by the DCC in collaboration with the WHO study coordination team. Investigators and study coordinators will randomly visit the field sites to observe study staff, supervisors and IOAs performing their activities. They will cross-check a small proportion of collected case report forms (CRFs) and share observations with supervisors and IOAs.

### External monitoring

The WHO study coordinating team will visit each site before the initiation of the study to assist in the preparation and planning of the site implementation of the protocol and study procedures. Due to restrictions on travel because of the COVID pandemic, virtual visits and the monthly online meeting will also be held. Country-based WHO monitors or other monitors will visit each site at least once or twice a year to monitor the progress and observe the procedures using a checklist to monitor clinical trials. It will include assessment and enrolment procedures, clinical practices such as counting respiratory rate, identifying danger signs, conducting pulse oximetry, and review other study procedures, and data management. Various logbooks will be checked. A proportion of completed electronic CRFs will be checked, and all SAE reporting forms will be reviewed. WHO monitors will also observe physician awareness and will meet with the regular hospital staff. In addition, they will make a home visit to those who are receiving treatment in the intervention arm and talk with the parents. When possible, the monitors will meet with the community groups’ members, ministry and district health officers, and programme managers at different levels. Recommendations arising from the site monitoring visits will be shared and discussed with the principal investigators and other project staff at the site office, and follow-up actions will be undertaken.

### Ethical considerations

The protocol has been approved by all the local institutional ethical review committees at the study sites and the WHO ethical review committee. Where required, national and regional / state approvals have been obtained. The safety of enrolled infants in this trial will be ensured by close monitoring and follow-up. The trial will follow CIOMS and GCP guidelines.

### Informed consent

Informed written consent will be obtained from parents / caregivers by a study nurse / physician at two stages, first at the time of screening / pre-enrolment and later at the time of enrolment, and will involve detailed verbal communication in the study participants’ native language to ensure comprehension of the trial and study procedures. If the parents/ caregivers are illiterate / unable to sign, then consent will be administered in the presence of a witness. All consent forms will be translated into local languages. Parents / caregivers of sick young infants will be provided basic information about the study and invited to consent for screening. Parents / caregivers of infants found to be eligible after the screening will be provided full information about the study and invited to have their infant participate in the study. The eligible infants will be enrolled if their parents provide informed consent and will be randomised to either one of the treatment arms according to the study approach. Illiterate parents / caregivers will be asked to give a thumbprint on the consent form; literate parents / caregivers will be requested to sign the consent form. Study staff will be trained in the study methodology, including obtaining consent.

### Patient safety

The study procedures and data collection will not pose any significant physical, psychological, social, legal or other kinds of risks to the participants. Clinical assessment and illness management, including the provision of drugs to be used in this study, are routinely used in clinical practice. Every enrolled subject will be followed up till the completion of treatment and assessed on day 15 of the enrolment. Poor clinical outcomes (except deaths) will receive an appropriate change of antibiotic therapy according to standard clinical practice. Those needing hospitalisation will be referred and admitted to the sub-district / district / tertiary care hospitals immediately for appropriate management as required. Other discomforts to caregivers might include a longer waiting time for data collection. However, the procedures will be explained in detail, and any queries by parents will be answered. The mobile phone number of the responsible study staff and study supervisors will be provided to all participants’ caregivers so that they could reach them if needed.

We will counsel and empower the mother / caregivers / families at enrolment, and all follow-up visits to 1) recognise danger signs or signs of illness and seek care and 2) know when to return to the hospital for follow-up care. We will train the mother / caregiver / family on the quantity, frequency and process of giving oral antibiotics at home. Systems will be in place in the hospital to provide emergency and rescue care in case of any adverse event or facilitate referral to a higher facility if required.

### Adverse events – documentation, reporting and response

Any adverse event that occurs after enrolment will be recorded on an adverse event reporting form by the treating nurse / physician. In case of an SAE, the study staff will contact the study supervisor and IOA. The IOA will document the SAE and convey the information to the study coordinator / investigator. An SAE, like death, anaphylactic reaction, severe diarrhoea, and a disseminated or severe rash will be reported to WHO within 48 hours of the occurrence. These cases will be (except, of course, the ones who unfortunately die) provided appropriate treatment and will be followed up. In case of other minor adverse effects, such as mild rash etc., the treatment will be continued. WHO will periodically report SAEs to the DSMB.

### Withdrawal from the study

Parents / caregivers can withdraw at any time after enrolment, and those who withdraw at any stage from the study will continue to receive free-of-charge standard treatment and treating physicians will follow them as per hospital standard procedures. These cases will be excluded from the per-protocol analysis but will be included in the intention-to-treat analysis.

### Non-enrolled cases

All non-enrolled cases who will be excluded during re-screening after 48 hours of initial screening and critically ill cases will be followed up on Day-15 after the initial screening to obtain information about their survival status. Consent will be obtained from them about collecting this information. The survival status of each non-enrolled infant will be collected either through a home / hospital visit (wherever the infant will be present) or by calling his / her parents / caregivers. This information will be used for the secondary analyses.

### Steering committee

This will comprise all Principal Investigators from study sites, consultants, donor representatives and WHO technical staff (secretariat). WHO will be responsible for organising Steering Committee meetings before study implementation, 9-12 months into the study and at the end of the study or when needed. This committee will be responsible for designing and implementing the study in a harmonised way. Study Principal Investigators will be responsible for contributing to the development of the research proposal, study manual, data management system, implementation of the intervention, outcome measurement and data collection, data analysis and interpretation and dissemination of results. All activities will be facilitated and supported by WHO. Every month, the sites will submit a brief status report to WHO. A formal progress report will be submitted by each site every year.

### Technical Advisory Group (TAG)

This group will be set up that will include three external experts in the field. The TAG members will serve in their capacity and will review the final research protocol for any major concerns before trial implementation. TAG members’ terms of reference also include review of the manual of operations, study forms and consent forms and advice on practical issues in implementing the trial in the field. WHO will serve as secretariat to this group and organise two meetings, one before the study starts and one after one year of study or when needed.

### Data safety monitoring board (DSMB)

An independent DSMB will be constituted to monitor the trial at regular intervals.

### Community and health sector engagement and dissemination of results

In the clinical settings where this study will be conducted, before its initiation, the local investigators will engage in dialogue with hospital health staff, ministry of health staff, community representatives, community-based organisations and non-governmental organisations working in the area to explain to them various aspects of this study.

Public health administrators will be part of the research teams and health care staff. The health facility administrators and professional health facilities linked with the study will also be sensitised about this research through personal contacts and sensitisation meetings. They will be informed that some patients with PSBI will be referred to tertiary care hospitals, and assistance with management will be needed for those children.

The potential audience for dissemination will be government officials, policymakers, academics, researchers, the local community and other voluntary organisations involved with community-based services. To reach this varied audience, a multipronged dissemination strategy will be required. We will invite the audience to dissemination seminars, which will be organised at the end of the project. For local communities, we will hold meetings with the community members and their leaders. We will publish the findings of these studies in peer-reviewed journals, with local researchers as the lead authors. We will submit abstracts at national and international conferences.

### Trial registration

The trial is registered at the International Standard Randomised Controlled Trial Number (ISRCTN) registry as ISRCTN16872570.

## DISCUSSION

The WHO recommends hospitalisation and treatment with injectable antibiotics for seven days for all young infants with signs of suspected clinical sepsis / PSBI [4]. Not all infants with these conditions have access to healthcare services and treatment [6,8,9,32,33]. Some sick young infants may not need a lengthy hospitalisation for seven days. The overburdened hospitals in LMICs could benefit from shorter hospitalisation for some sick infants with PSBI signs, which will facilitate more sick young infants getting treatment for PSBI. It will allow the hospitals to focus more on critically ill infants who need standardised inpatient care and treatment needed to reduce neonatal mortality. It will also help families with limited resources who cannot stay for longer periods in a hospital, which entails a loss of wage, extra costs for transport and food for the patient attendants, and a lack of attendants for the care of other children back home [34-37].

We will compare the proportion of poor clinical outcomes in hospitalised young infants with moderate-risk mortality signs with switch therapy to an oral antibiotic after initial hospitalisation in those. We have cautiously and pragmatically designed this multi-centre trial to treat young infants with moderate mortality-risk signs of PSBI safely and effectively to achieve generalisable results. The outcome assessment will be conducted periodically by independent assessors at home for intervention arm infants or in the hospital in case of admitted infants in the control arm. The enrolled infants will be diagnosed and treated based on clinical signs recommended in the WHO PSBI guidelines [3-5], without microbiological confirmation of bacterial aetiology. However, we will use a well-established objective marker like CRP to identify clinically stable infants for randomisation.

Our study has several strengths. First, a multi-centre trial in several African and Asian sites with a fairly large sample size from diverse populations will provide precision and generalisability. Second, this trial is being conducted in collaboration with the local governments, which will facilitate future policy adoption if required. Third, standardised training, supervision, oversight, and monitoring will be undertaken to ensure quality, consistency, harmonised trial procedures and implementation. Documentation of treatment and follow-up will be carried out by trained study staff. Investigators will ensure that the protocol and standard operating procedures are followed, data is accurately collected, and the highest level of safety is provided. Outcome assessment by an independent assessor will minimise ascertainment bias. Fourth, enrolment of self-referred babies to the hospitals minimises selection bias. Finally, in addition to regular site monitoring visits by study staff and investigators, the WHO monitoring and coordinating team in Geneva will be engaged to assist with close external monitoring of the study, including regular site monitoring visits to assess compliance with human subjects, other research regulations and guidelines, adherence to the study protocol and procedures, quality and accuracy of data collected, and quality of care and child safety.

The study has some limitations. First, enrolled children will be clinically diagnosed using WHO PSBI guidelines without using microbiological and / or radiological methods. While microbiological and / or other diagnostic aids may add improved specificity to the clinical diagnosis of PSBI, good quality microbiological services are a challenge in most hospitals in LMICs and would limit the generalisability of the study. Second, blinding of intervention therapy is not possible. Third, there may be an element of subjectivity involved in the assessment of poor clinical outcomes based on the presence or absence of clinical signs. To reduce this bias, independent assessors will conduct outcome assessments in both intervention and control arms. Finally, a question may arise about the generalisability of the results because we will be strengthening hospital-based care in the study facilities from an ethical standpoint, which may be different from other facilities that will not get such support.

## CONCLUSIONS

If the results show that young infants with moderate-mortality risk PSBI signs can be safely and effectively treated with shorter hospitalisation and switch therapy, it may substantially increase access to treatment for families with limited means who cannot afford to be away from their homes and work for extended periods. It may also reduce the health system costs (human and materials) and allow the overburdened hospitals to focus on critically ill young infants. This evidence will contribute toward making a case for reviewing the WHO PSBI management [3,4,5].

## References

[1] United Nations Inter-agency Group for Child Mortality Estimation (UN IGME). Levels and Trends in Child Mortality: Report 2022. New York: United Nations Children’s Fund; 2023. Available: https://data.unicef.org/resources/levels-and-trends-in-child-mortality/. Accessed: 8 February 2023.

[2] Alliance for Maternal and Newborn Health Improvement (AMANHI) mortality study group Population-based rates, timing, and causes of maternal deaths, stillbirths, and neonatal deaths in south Asia and sub-Saharan Africa: a multi-country prospective cohort study. Lancet Glob Health. 2018;6:e1297-308. 10.1016/S2214-109X(18)30385-1 30361107 PMC6227247

[3] World Health Organization. Integrated Management of Childhood Illness: management of the sick young infant aged up to 2 months. IMCI chart booklet. Geneva, Switzerland: WHO; 2019 Available: https://www.who.int/maternal_child_adolescent/documents/management-sick-young-infant-0-2-months/en/. Accessed: 8 February 2023.

[4] World Health Organization. Pocket book of hospital care for children: guidelines for the management of common childhood illnesses. 2nd ed. Switzerland: World Health Organization; 2013. Available: https://apps.who.int/iris/bitstream/handle/10665/81170/9789241548373_eng.pdf?sequence=1. Accessed: 8 February 2023. 24006557

[5] World Health Organization. Guideline: Managing possible serious bacterial infection in young infants when referral is not feasible. Switzerland: WHO; 2015. Available: https://www.who.int/maternal_child_adolescent/documents/bacterial-infection-infants/en/. Accessed: 8 February 2023. 26447263

[6] Tshefu A Lokangaka A Ngaima S Engmann C Esamai F Gisore P Simplified antibiotic regimens compared with injectable procaine benzylpenicillin plus gentamicin for treatment of neonates and young infants with clinical signs of possible serious bacterial infection when referral is not possible: a randomised, open-label, equivalence trial. Lancet. 2015;385:1767-76. 10.1016/S0140-6736(14)62284-4 25842221

[7] Tshefu A Lokangaka A Ngaima S Engmann C Esamai F Gisore P Oral amoxicillin compared with injectable procaine benzylpenicillin plus gentamicin for treatment of neonates and young infants with fast breathing when referral is not possible: a randomised, open-label, equivalence trial. Lancet. 2015;385:1758-66. 10.1016/S0140-6736(14)62285-6 25842223

[8] Baqui AH Saha SK Ahmed AS Shahidullah M Quasem I Roth DE Safety and efficacy of alternative antibiotic regimens compared with 7 day injectable procaine benzylpenicillin and gentamicin for outpatient treatment of neonates and young infants with clinical signs of severe infection when referral is not possible: a randomised, open-label, equivalence trial. Lancet Glob Health. 2015;3:e279-87. 10.1016/S2214-109X(14)70347-X 25841891

[9] Mir F Nisar I Tikmani SS Baloch B Shakoor S Jehan F Simplified antibiotic regimens for treatment of clinical severe infection in the outpatient setting when referral is not possible for young infants in Pakistan (Simplified Antibiotic Therapy Trial [SATT]): a randomised, open-label, equivalence trial. Lancet Glob Health. 2017;5:e177-85. 10.1016/S2214-109X(16)30335-7 27988146 PMC5250591

[10] Wammanda RD Adamu SA Joshua HD Nisar YB Qazi SA Aboubaker S Implementation of the WHO guideline on treatment of young infants with signs of possible serious bacterial infection when hospital referral is not feasible in rural Zaria, Nigeria: Challenges and solutions. PLoS One. 2020;15:e0228718. 10.1371/journal.pone.0228718 32155155 PMC7064229

[11] Guenther T Mopiwa G Nsona H Qazi S Makuluni R Fundani CB Feasibility of implementing the World Health Organization case management guideline for possible serious bacterial infection among young infants in Ntcheu district, Malawi. PloS One. 2020;15:e0229248. 10.1371/journal.pone.0229248 32287262 PMC7156088

[12] Rahman AE Herrera S Rubayet S Banik G Hasan R Ahsan Z Managing possible serious bacterial infection of young infants where referral is not possible: Lessons from the early implementation experience in Kushtia District learning laboratory, Bangladesh. PLoS One. 2020;15:e0232675. 10.1371/journal.pone.0232675 32392209 PMC7213695

[13] Awasthi S Kesarwani N Verma RK Agarwal GG Tewari LS Mishra RK Identification and management of young infants with possible serious bacterial infection where referral was not feasible in rural Lucknow district of Uttar Pradesh, India: An implementation research. PLoS One. 2020;15:e0234212. 10.1371/journal.pone.0234212 32497092 PMC7272098

[14] Roy S Patil R Apte A Thibe K Dhongade A Pawar B Feasibility of implementation of simplified management of young infants with possible serious bacterial infection when referral is not feasible in tribal areas of Pune district, Maharashtra, India. PLoS One. 2020;15:e0236355. 10.1371/journal.pone.0236355 32833993 PMC7446882

[15] Goyal N Rongsen-Chandola T Sood M Sinha B Kumar A Qazi SA Management of possible serious bacterial infection in young infants closer to home when referral is not feasible: Lessons from implementation research in Himachal Pradesh, India. PLoS One. 2020;15:e0243724. 10.1371/journal.pone.0243724 33351810 PMC7755274

[16] Leul A Hailu T Abraham L Bayray A Terefe W Godefay H Innovative approach for potential scale-up to jump-start simplified management of sick young infants with possible serious bacterial infection when a referral is not feasible: Findings from implementation research. PLoS One. 2021;16:e0244192. 10.1371/journal.pone.0244192 33544712 PMC7864440

[17] Mukhopadhyay R Arora NK Sharma PK Dalpath S Limbu P Kataria G Lessons from implementation research on community management of Possible Serious Bacterial Infection (PSBI) in young infants (0-59 days), when the referral is not feasible in Palwal district of Haryana, India. PLoS One. 2021;16:e0252700. 10.1371/journal.pone.0252700 34234352 PMC8279773

[18] Ayede AI Ashubu OO Fowobaje KR Aboubaker S Nisar YB Qazi SA Management of possible serious bacterial infection in young infants where referral is not possible in the context of existing health system structure in Ibadan, South-west Nigeria. PLoS One. 2021;16:e0248720. 10.1371/journal.pone.0248720 33784321 PMC8009401

[19] Applegate JA Ahmed S Khan MA Alam S Kabir N Islam M Early implementation of guidelines for managing young infants with possible serious bacterial infection in Bangladesh. BMJ Glob Health. 2019;4:e001643. 10.1136/bmjgh-2019-001643 31803507 PMC6882554

[20] Berhane M Girma T Tesfaye W Jibat N Abera M Abrahim S Implementation research on management of sick young infants with possible serious bacterial infection when referral is not possible in Jimma Zone, Ethiopia: challenges and solutions. PLoS One. 2021;16:e0255210. 10.1371/journal.pone.0255210 34370744 PMC8351942

[21] Ariff S Soofi SB Suhag Z Chanar S Bhura M Dahar Z Implementation research to increase treatment coverage of possible serious bacterial infections in young infants when a referral is not feasible: lessons learnt. Journal of public health (Oxf.). 2023 10.1093/pubmed/fdab409 35138390 PMC10017086

[22] Sands K Carvalho MJ Portal E Thomson K Dyer C Akpulu C Characterisation of antimicrobial-resistant Gram-negative bacteria that cause neonatal sepsis in seven low- and middle-income countries. Nat Microbiol. 2021;6:512-23. 10.1038/s41564-021-00870-7 33782558 PMC8007471

[23] Dramowski A Madide A Bekker A Neonatal nosocomial bloodstream infections at a referral hospital in a middle-income country: burden, pathogens, antimicrobial resistance and mortality. Paediatr Int Child Health. 2015;35:265-72. 10.1179/2046905515Y.0000000029 25940506

[24] Maoulainine FM Elidrissi NS Chkil G Abba F Soraa N Chabaa L [Epidemiology of nosocomial bacterial infection in a neonatal intensive care unit in Morocco]. Arch Pediatr. 2014;21:938-43. 10.1016/j.arcped.2014.04.033 24993147

[25] Shahunja KM Ahmed T Faruque AS Shahid AS Das SK Shahrin L Experience With Nosocomial Infection in Children Under 5 Treated in an Urban Diarrheal Treatment Center in Bangladesh. Glob Pediatr Health. 2016;3: 2333794x16634267. 10.1177/2333794X16634267 27336005 PMC4905154

[26] Nisar YB Tshefu A Longombe AL Esamai F Marete I Ayede AI Clinical signs of possible serious infection and associated mortality among young infants presenting at first-level health facilities. PLoS One. 2021;16:e0253110. 10.1371/journal.pone.0253110 34191832 PMC8244884

[27] Sivanandan S Soraisham AS Swarnam K Choice and duration of antimicrobial therapy for neonatal sepsis and meningitis. Int J Pediatr. 2011;2011:712150. 10.1155/2011/712150 22164179 PMC3228399

[28] National Institute for Health and Care Excellence (NICE). NICE guideline: Neonatal infection: antibiotics for prevention and treatment. NICE; 2021. Available: https://www.nice.org.uk/guidance/ng195. Accessed: 8 February 2023. 34133110

[29] Pantell RH Roberts KB Adams WG Dreyer BP Kuppermann N O’Leary ST Clinical Practice Guideline: Evaluation and Management of Well-Appearing Febrile Infants 8 to 60 Days Old. Pediatrics. 2021;148. 10.1542/peds.2021-052228 34281996

[30] World Health Organization. WHO guidelines on drawing blood: best practices in phlebotomy. Geneva, Switzerland: WHO; 2010. 23741774

[31] International Standard Randomised Controlled Trial Number (ICRCTN) Registry. Optimal place of treatment for young infants aged less than 2 months with any one low-mortality-risk sign of possible serious bacterial infection. ISRCTN44033252. 10.1186/ISRCTN44033252. Accessed: 8 February 2023. 10.1186/ISRCTN44033252 PMC10346131 37449353

[32] Bang AT Bang RA Baitule SB Reddy MH Deshmukh MD Effect of home-based neonatal care and management of sepsis on neonatal mortality: field trial in rural India. Lancet. 1999;354:1955-61. 10.1016/S0140-6736(99)03046-9 10622298

[33] Seale AC Blencowe H Manu AA Nair H Bahl R Qazi SA Estimates of possible severe bacterial infection in neonates in sub-Saharan Africa, south Asia, and Latin America for 2012: a systematic review and meta-analysis. Lancet Infect Dis. 2014;14:731-41. 10.1016/S1473-3099(14)70804-7 24974250 PMC4123782

[34] Applegate JA Ahmed S Harrison M Callaghan-Koru J Mousumi M Begum N Caregiver acceptability of the guidelines for managing young infants with possible serious bacterial infections (PSBI) in primary care facilities in rural Bangladesh. PLoS One. 2020;15:e0231490. 10.1371/journal.pone.0231490 32287286 PMC7156040

[35] Teklu AM Litch JA Tesfahun A Wolka E Tuamay BD Gidey H Referral systems for preterm, low birth weight, and sick newborns in Ethiopia: a qualitative assessment. BMC Pediatr. 2020;20:409. 10.1186/s12887-020-02311-6 32861246 PMC7456368

[36] Kozuki N Guenther T Vaz L Moran A Soofi SB Kayemba CN A systematic review of community-to-facility neonatal referral completion rates in Africa and Asia. BMC Public Health. 2015;15:989. 10.1186/s12889-015-2330-0 26419934 PMC4589085

[37] Owais A Sultana S Stein AD Bashir NH Awaldad R Zaidi AK Why do families of sick newborns accept hospital care? A community-based cohort study in Karachi, Pakistan. J Perinatol. 2011;31:586-92. 10.1038/jp.2010.191 21273989 PMC3152606

